# Nootkatone Mitigated Melamine-Evoked Hepatotoxicity by Featuring Oxidative Stress and Inflammation Interconnected Mechanisms: In Vivo and In Silico Approaches

**DOI:** 10.3390/toxics11090784

**Published:** 2023-09-15

**Authors:** Ola A. Habotta, Ahmed Abdeen, Ali B. Roomi, Afnan I. Elgndy, Safwa M. Sorour, Maha H. Morsi, Kamal M. Kamal, Samah F. Ibrahim, Doaa Abdelrahaman, Liana Fericean, Ioan Banatean-Dunea, Heba I. Ghamry, Mohammad El-Nablaway, Reem T. Atawia, Dania Abdelhady

**Affiliations:** 1 Department of Forensic Medicine and Toxicology, Faculty of Veterinary Medicine, Mansoura University, Mansoura 35516, Egypt; 2 Department of Forensic Medicine and Toxicology, Faculty of Veterinary Medicine, Benha University, Toukh 13736, Egypt; 3 Department of Quality Assurance, University of Thi-Qar, Thi-Qar, Nasiriyah 64001, Iraq; 4 Department of Medical Laboratory, College of Health and Medical Technology, National University of Science and Technology, Thi-Qar, Nasiriyah 64001, Iraq; 5 Department of Physiology, Faculty of Medicine, Benha University, Benha 13518, Egypt; 6 Department of Pharmacology, Faculty of Medicine, Benha University, Benha 13518, Egypt; 7 Department of Clinical and Chemical Pathology, Faculty of Applied Health Sciences Technology, Misr University for Science and Technology, Giza 3236101, Egypt; 8 Department of Anatomy and Embryology, Faculty of Medicine, Benha University, Benha 13518, Egypt; 9 Department of Clinical Sciences, College of Medicine, Princess Nourah bint Abdulrahman University, P.O. Box 84428, Riyadh 11671, Saudi Arabia; 10 Department of Basic Sciences, Faculty of Medicine, Al-Azhar University, Cairo 11751, Egypt; 11 Department of Biology and Plant Protection, Faculty of Agriculture, University of Life Sciences “King Michael I” from Timișoara, Calea Aradului 119, CUI 3487181, 300645 Timisoara, Romania; 12 Nutrition and Food Sciences, Department of Home Economics, Faculty of Home Economics, King Khalid University, P.O. Box 960, Abha 61421, Saudi Arabia; 13 Department of Medical Biochemistry, Faculty of Medicine, Mansoura University, Mansoura 35516, Egypt; 14 Department of Basic Medical Sciences, College of Medicine, AlMaarefa University, P.O. Box 71666, Riyadh 11597, Saudi Arabia; 15 Department of Pharmacology and Toxicology, Faculty of Pharmacy, Ain Shams University, Cairo 11566, Egypt; 16 Department of Pharmaceutical Sciences, College of Pharmacy, Southwestern Oklahoma State University, Weatherford, OK 73096, USA

**Keywords:** melamine, nootkatone, liver injury, oxidative stress, Nrf2, NF-κB, molecular docking

## Abstract

Melamine (ML) is a common environmental contaminant, commonly used in food fraud, representing a serious health hazard and jeopardizing human and animal health. Recently, nootkatone (NK), a naturally occurring sesquiterpenoid, has garnered considerable attention due to its potential therapeutic advantages. We investigated the potential mechanisms underlying the protective effects of NK against ML-induced liver injury in rats. Five groups were utilized: control, ML, NK10, ML-NK5, and ML-NK10. ML induced substantial hepatotoxicity, including considerable alterations in biochemical parameters and histology. The oxidative distress triggered by ML increased the generation of malondialdehyde (MDA) and nitric oxide (NO) and decreased levels of reduced glutathione (GSH), catalase (CAT), and superoxide dismutase (SOD) activities. In addition, decreased expression of nuclear factor-erythroid 2-related factor 2 (Nrf2) and increased nuclear factor kappa beta (NF-κB) expression levels were observed in hepatocytes, which indicated the occurrence of inflammatory changes following ML exposure. These alterations were alleviated by NK supplementation in a dose-dependent manner. The data revealed that the favorable effects of NK were attributed, at least in part, to its antioxidant and anti-inflammatory properties. Moreover, our results were supported by molecular docking studies that revealed a good fit and interactions between NK and antioxidant enzymes. Thus, the current study demonstrated that NK is a potential new food additive for the prevention or treatment of ML-induced toxicity.

## 1. Introduction

Melamine (ML; 2,4,6-triamino-1,3,5-triazine) is a triazine heterocyclic chemical, frequently employed for the production of resins, plastics, enamel dyes, commercial filters, glues, dishware, and kitchenware [1,2,3,4]. Furthermore, this organic base contains 66% nitrogen, and has been added to food for human and animal consumption; the latter has been shown to fraudulently boost the apparent protein content for commercial gain [5]. Several studies have demonstrated contamination of various types of foods by ML, including fruits, baby formula, milk, yogurt, cheese, butter, eggs, processed meat, and bread [6,7], causing considerable concern, because it jeopardizes human and animal health [8]. Zhang and colleagues [9] found that ML was able to induce DNA damage in sperm with a significant increase in sperm abnormality rates in exposed mice. ML residues accumulate in different organs after intravenous administration, causing toxic effects in the brain, spleen, bladder, and kidney [10]. ML also damages the liver, the chief site for detoxification and elimination in the body [10]. Previous investigations have reported that ML induced significant alterations in hepatic histoarchitecture and elevated liver function markers, as well as the induction of oxidative stress, inflammation, and apoptotic changes [2,11,12].

Many compelling publications have supported the presence of oxidative stress following ML exposure, primarily due to the depletion of the intracellular antioxidant defense systems and the uncontrolled formation of reactive oxygen species (ROS) such as superoxide anion, O_2_^•–^; hydrogen peroxide, H_2_O_2_; and hydroxyl radical, OH^•^; as well as reactive nitrogen species such as nitric oxide (NO) [11,13]. These events cause tissue damage, lipid peroxidation (LPO), protein cross-linking, and DNA oxidation [12,14,15]. Another proposed mechanism following long-term exposure to ML is the enhancement of pro-inflammatory and pro-fibrotic markers [16]. Currently, there is no specific treatment for ML toxicity and treatment depends on controlling the ML-induced damage, removal of renal stones, and dialysis, if needed [17]. Consequently, antioxidant supplementation might be an effective therapeutic strategy to repair ML-induced tissue damage and enhance tissue renewal.

Antioxidants of plant origin have garnered global attention recently and are frequently used due to their phytochemical content [18]. Among them, nootkatone (NK; C_15_H_22_O) is a sesquiterpenoid extracted from several plants including grapefruit and rhizomes of *Cyperus rotundas*, as well as essential oil derived from citrus [19]. Due to its pleasant aroma, this compound has been commercially employed in the chemical, food, and cosmetic industries [20]. NK has tremendous pharmacological properties, including antioxidant, anti-inflammatory, and antiapoptotic activities [19,21]. In addition, an increasing body of evidence supports the potential protective effects of NK against certain drugs and environmental toxicants such as isoproterenol [22], carbon tetrachloride [23,24], rotenone [25], D-galactosamine [26], lipopolysaccharide [27], and water pipe smoking [28].

Therefore, a literature review revealed that even though numerous studies have been performed regarding ML toxicity, research focused on ML-induced liver injury and its alleviation is minimal. Therefore, this study evaluated whether NK exhibited a modulatory effect on ML-triggered oxidative stress and inflammatory liver damage. Hepatic biochemical parameters, oxidative status, histological alterations, and expression of nuclear factor erythroid 2-related factor 2 (Nrf2) and nuclear factor kappa beta (NF-κB) were investigated.

## 2. Materials and Methods

### 2.1. Chemicals

Melamine and nootkatone were obtained from Sigma Chemical Company (St. Louis, MO, USA). The analytical kits used to assay the biochemical parameters and oxidative cascade markers were purchased from Bio-diagnostics Co., Giza, Egypt.

### 2.2. Animals and Ethical Endorsement

Male Wister albino rats weighing 150–170 g were used to complete this experimental protocol. Rats were procured from the Medical Experimental Research Center of Mansoura University (MERC) for experimental research, Faculty of Veterinary Medicine, Mansoura, Egypt. Before the trial, the rats were confined in comfortable, standard hygienic conditions for two weeks (temperature: ~25 °C, humidity: 50–60%). During the trial, all rats were fed a standard baseline diet and had unrestricted access to water. 

### 2.3. Experimental Modeling 

After being acclimated, animals were sorted into five equivalent groups (five rats/group). The control group received food and water *ad libitum* without any treatment. The ML group was the positive toxic group, in which rats received 700 mg/kg body weight ML [11]. In the NK group, rats were treated with NK10 (10 mg/kg body weight) [22]. In the ML-NK5 group, rats were given a low dose of both ML and NK, of 5 mg/kg, while in the ML-NK10 group, rats received ML and NK in high doses of 10 mg/kg (ML was given in the same regimen used with the ML group). All treatments were given orally, once a day for 28 successive days.

### 2.4. Sampling

At the end of the trial, the rats were euthanized by intraperitoneal injection of a xylazine and ketamine mixture (1:1 *v*/*v*; 0.15 mL/100 g body weight). Blood specimens were gathered from the heart of each animal. The serum was collected after centrifugation of the coagulated blood at 2000× *g* for 10 min and preserved at −20 °C until further analysis. Immediately following blood collection, the liver was rapidly extracted, washed in ice-cold physiological saline, and sliced into several pieces. One piece was placed in neutral buffered formalin (10%) for subsequent histopathological evaluation. The other fresh tissue samples were preserved at −80 °C for oxidative cascade marker investigation.

### 2.5. Estimation of Liver Biomarkers

Serum was used to estimate the liver biochemical markers (ALP, AST, and ALT) using kits from Bio-diagnostics Co. The analysis of all markers was performed according to the manufacturer’s directions.

### 2.6. Liver Homogenate Preparation 

Liver homogenates from each rat were prepared by centrifuging the tissue samples at 8000 rpm for 15 min at 4 °C in 400 mL of phosphate-buffered saline (PBS). The supernatants were collected in cold Eppendorf tubes for enzymatic analyses. 

### 2.7. Estimation of the Hepatic Oxidative State

The non-enzymatic oxidative markers (MDA, GSH, and NO) and the enzymatic oxidative marker (CAT) were purchased from Bio-diagnostics Co, and measured according to the manufacturers’ guidelines.

### 2.8. Histoarchitecture Analysis

Formalin-fixed liver samples were dehydrated in a graded alcohol series. Subsequently, xylene clearing was performed followed by routine paraffin embedding. The tissue was cut into 5 μm sections and stained with hematoxylin and eosin (H&E) for histological evaluation. Representative images were captured using an integrated digital scanning camera system (DM300, Leica, Germany).

### 2.9. Immunohistochemical Examination

Following the manufacturer’s directions, immunohistochemical staining was used to evaluate the hepatic expression of NF-κB and Nrf2. Liver sections (4 mm) on microscope slides were incubated in 0.3% hydrogen peroxide for 20 min to prevent endogenous peroxidase activity. The sections were blocked for 30 min with 2% bovine serum albumin (BSA) for 10 min before incubation in a water bath at 100 °C. Then, the sections were incubated overnight at 4 °C in rat antibodies directed against a rat-targeted antigen. Subsequently, the sections were washed and then incubated with horseradish peroxidase (HRP) secondary antibody at 37 °C for 1 h. Then, the sections were exposed to a diaminobenzidine (DAB) working solution for 4 min and counterstained with Mayer’s hematoxylin. Finally, the sections were evaluated and photographed. Images were taken using a Nikon integrated digital imaging system (Eclipse E200-LED, Nikon, Tokyo, Japan), at an original magnification of ×400.

### 2.10. Molecular Docking

ML was docked with rat superoxide dismutase (SOD1, SOD2, SOD3), catalase (CAT), glutathione peroxidase-1 (GPx-1), glutamate-cysteine ligase catalytic subunit (GCLC), glutathione reductase (GR), and glutathione synthase (GS). NK was docked against tumor necrosis factor receptor superfamily member 1A (TNFRSF1A), tumor necrosis factor receptor superfamily member 1B (TNFRSF1B), transforming growth factor beta-activated kinase 1 (TAK1), inhibitor of nuclear factor kappa-B kinase subunit beta (IKKB), interleukin-1 receptor type 1 (IL-1R1), interleukin-1 receptor type 1 (IL-1R2), caspase-3, and inducible nitric oxide synthase (iNOS). The three-dimensional structures of target proteins were retrieved from UniProt (https://www.uniprot.org/; accessed on 15 June 2023) and AlphaFold (https://alphafold.ebi.ac.uk/; accessed on 15 June 2023) protein structure databases. Proteins were prepared for docking using Molecular Operating Environment software (MOE 2022.02, Chemical Computing Group, Montreal, QC, Canada). In addition, the three-dimensional structures of ML and NK were retrieved from the PubChem (https://pubchem.ncbi.nlm.nih.gov/; accessed on 15 June 2023) database. Furthermore, the molecular docking, protein–ligand interactions, and visualization were carried out using MOE software (https://www.chemcomp.com/ accessed on 15 June 2023).

### 2.11. Statistical Data

One-way analysis of variance (ANOVA) was used to analyze the results, and differences between the groups were revealed using Duncan’s post-hoc multiple tests. All values were judged to be statistically significant at *p* ≤ 0.05 and expressed as means ± standard error of the mean (SE). GraphPad Prism version 8 was used for data analysis and the generation of column charts. Multivariate principal component analysis (PCA), the variable importance in projection (VIP) score, and clustering heatmaps were created using MetaboAnalyst software (version 0.5, developed by the XiaLab at McGill University, Montreal, QC, Canada).

## 3. Results

### 3.1. Effect of ML and/or NK Treatment on Liver Biochemical Parameters 

**Figure 1:**
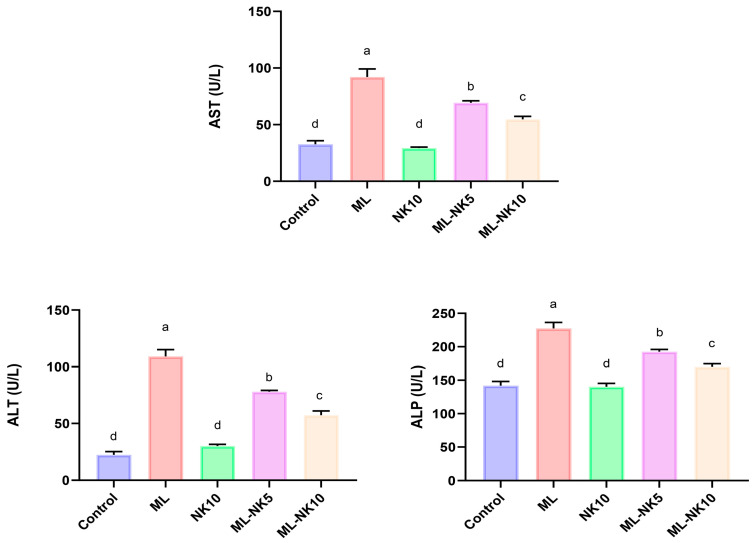
Bar plots of liver parameter levels with ML and/or NK exposure (*n* = 5). ALT, alanine aminotransferase; ALP, alkaline phosphatase; AST, aspartate aminotransferase. Control, nootkatone (NK10), melamine (ML), ML-NK5 combination, and ML-NK10 combination. Significant differences existed across groups with different letters (*p* ≤ 0.05).

As shown in Figure 1, ML treatment resulted in notable disruptions in liver functions, as indicated by a substantial increase in liver enzymes (AST and ALT) and ALP activity compared to control rats. On the other hand, preconditioning with a low dose of NK significantly attenuated the ML-induced injuries in liver tissue. Remarkably, administering a high dose of NK exerted noticeable amelioration of those parameters. These data suggested a dose-dependent change in ML-treated animals when co-administrated with different doses of NK. 

### 3.2. Effect of ML and/or NK Treatment on Oxidative Cascade and Lipid Peroxidation 

**Figure 2:**
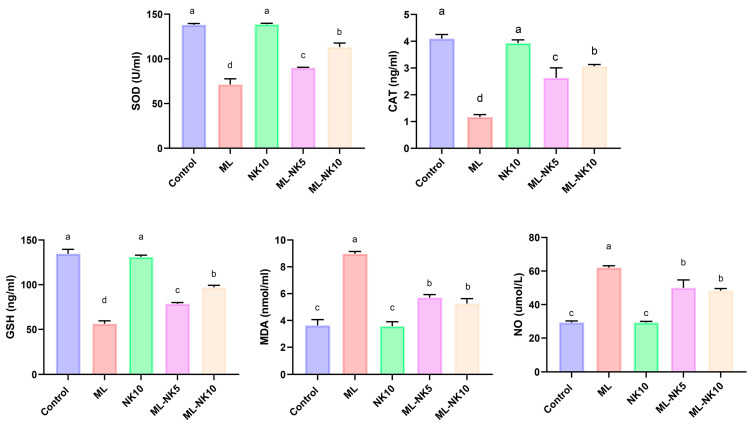
Bar plots of liver tissue oxidative biomarker alterations with ML and/or NK exposure (*n* = 5). CAT, catalase; MDA, malondialdehyde; NO, nitric oxide; GSH, reduced glutathione, SOD; superoxide dismutase. Control, nootkatone (NK10), melamine (ML), ML-NK5 combination, and ML-NK10 combination. Significant differences existed across groups with different letters (*p* ≤ 0.05).

The lipid peroxidation (MDA) index and oxidative status (NO, CAT, SOD, and GSH) following ML and/or NK administration are presented in Figure 2. ML treatment prompted considerable oxidative stress, as indicated by a drastic reduction in CAT and SOD activity as well as the level of GSH, along with increased MDA and NO levels, compared to controls. However, rats co-administrated ML and NK at high and low doses exhibited significant elevations in CAT and SOD activities and GSH levels, as well as a reduction in MDA and NO levels. An improvement in the oxidative state appeared in the ML-NK10 group compared to the ML-NK5 group in the CAT, SOD, and GSH expression levels. The MDA and NO levels exhibited no significant differences between the ML-NK5 and ML-NK10 groups (Figure 2). 

### 3.3. Liver Histopathology following ML and/or NK Treatment 

**Figure 3:**
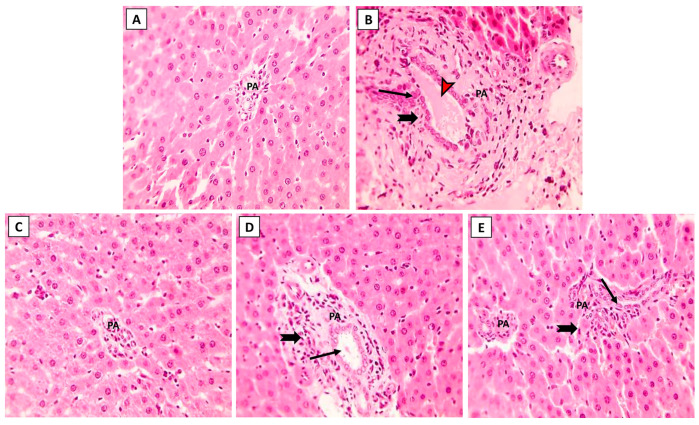
The effects of the dose-dependent NK co-treatment with ML exposure on the liver histoarchitecture. (**A**): Control and (**C**): NK10 groups exhibit normal liver histological patterns. (**B**): ML-exposed rat exhibits notable portal fibrosis coupled with prominent inflammatory cell extravasation (thick, black arrows), congested portal blood vessels (red, arrows), and dilation of biliary tracts with proliferation of the lining epithelium (thin, black arrows). The ML-NK5 and ML-NK10 groups display dose-dependent improvement in the liver histological features ranging from limited portal fibrosis with sparse inflammatory infiltration (thick, black arrows), and minimal biliary changes (thin, black arrows) (**D**) to strikingly nearly normal hepatic lobules (**E**). (PA, portal artery; H&E ×400).

The hepatic histoarchitecture of the control and NK groups revealed normal hepatic cords radially arranged around central veins and normal portal areas and sinusoids (Figure 3A,C). Hepatic sections from the ML group showed marked portal fibrosis with disruptions in the radial arrangement of the hepatocyte cords. Marked infiltration of inflammatory cells was observed in the hepatic lobules. Congested portal blood vessels, sinusoidal enlargement, and also dilation and proliferation of biliary epithelium were observed (Figure 3B). Sections from the ML-NK5 group demonstrated limited portal fibrosis with sparse inflammatory infiltration. In addition, there was decreased dilation and proliferation of the biliary tracts (Figure 3D). Co-treatment of rats with ML and NK10 resulted in strikingly nearly normal hepatic lobules with minimal inflammatory cell infiltration in the portal areas (Figure 3E). 

### 3.4. Nrf2 and NF-κB Expression in Hepatic Tissue

**Figure 4:**
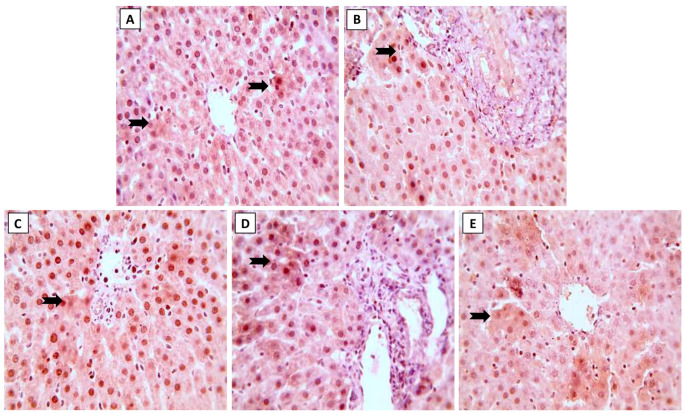
The effects of the dose-dependent concurrent NK treatment with ML exposure on Nrf2 expression in liver tissue. (**A**): Control and (**C**): NK groups display positive expression of Nrf2. (**B**): ML exposure dramatically downregulated the Nrf2 expression. (**D**): ML-NK5 and (**E**): ML-NK10 treatment revealed modestly and remarkably increased Nrf2 expression, respectively. The positive Nrf2 expression is indicated by the brown color of the hepatocyte cytoplasm (thick, black arrows).

**Figure 5:**
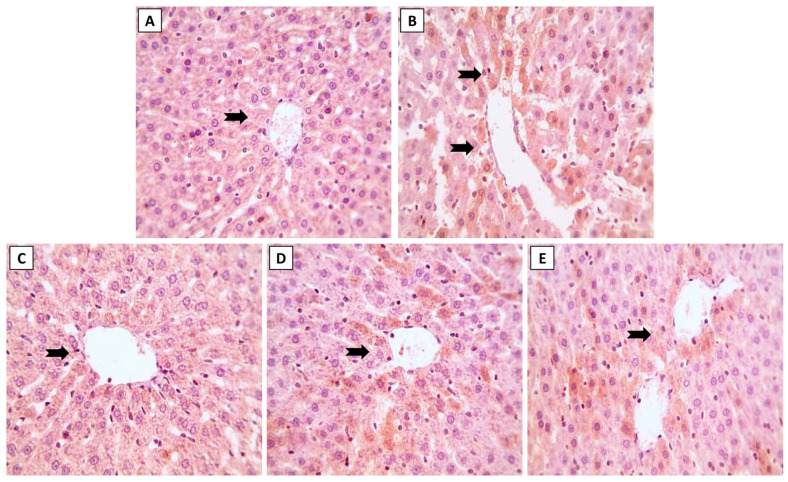
The effects of dose-dependent NK co-treatment with ML exposure on NF-κB expression in liver tissue. (**A**): Control and (**C**): NK groups reveal minimal expression of NF-κB. (**B**): ML-exposed tissue shows distinctly up-regulated NF-κB expression. (**D**): ML-NK5 and (**E**): ML-NK10 groups revealed modest and extremely low NF-κB expression, respectively. The positive NF-κB expression is indicated by the brown color of the hepatocyte cytoplasm (thick, black arrows).

Expression of Nrf2 and NF-κB in liver sections following treatment by ML and/or NK are displayed in Figure 4 and Figure 5, respectively. Immunohistochemical staining of liver sections showed changes in oxidative stress and inflammation-related markers with exposure to ML, as shown by the reduced expression of Nrf2 in hepatocytes (Figure 4B). On the other hand, we spotted modest (Figure 4D) and increased (Figure 4E) Nrf2 expression when animals were co-supplemented with 5 and 10 mg/kg of NK, respectively.

In addition, immunohistochemical evaluation of the liver sections revealed changes in the inflammatory state of the hepatic tissue after ML exposure, with up-regulated NF-κB expression (Figure 5B). In contrast to the ML-treated rats, we noticed modest (Figure 5D) and minimal (Figure 5E) NF-κB expression when rats were simultaneously treated with NK5 and NK10, respectively. These observations confirmed the hypothesis of dose-dependent improvement of ML-mediated hepatic damage, as elucidated by the remarkable changes observed in the immunological assessment when the rats were treated with high doses of NK (10 mg/kg).

### 3.5. Hierarchical Clustering Heatmap, PCA, and VIP Scores

**Figure 6:**
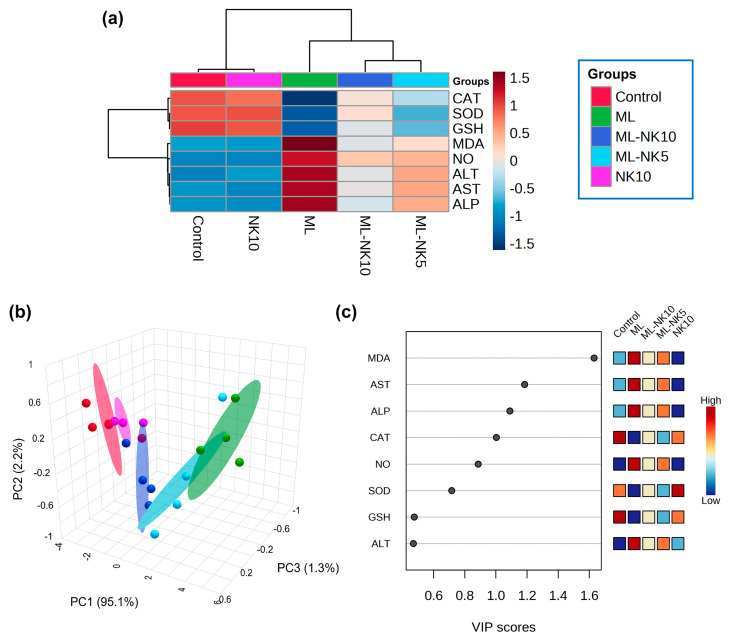
Multivariate analyses of ML and/or NK treatment. (**A**) hierarchical clustering heatmap reveals intuitive visualization of all data sets. Each colored cell on the map reflects the concentration levels of measured variables, with varying averages in rows and different treatment groups in columns. Dark red indicates the highest value and blue indicates the lowest value on the gradation scale. (**B**) The five experimental groups (Control, NK, ML, ML-NK5, and ML-NK10) were identified using a 3D score plot of PCA. Proportion values specified on the axes represent the contribution rates of PC1 (95.1%), PC2 (2.2%), and PC3 (1.3%) to the overall number of variations. (**C**) The variable importance in projection (VIP) shows the contribution intensity as a colored scale with the greatest value in red and the lowest value in blue. The colored boxes on the right show the proportional concentrations of the relevant measured parameters in each study group.

The clustering heatmap depicted in Figure 6A exemplifies a clear visual representation of all data groups that highlight the distinct disparities present in the levels of all measured parameters with respect to ML toxicity compared to the other groups. These findings imply that the ML-exposed animals incurred more injury than the other groups.

PCA was implemented to analyze the correlations between different treatments and variables. Based on PCA, all variables were sorted into three principal dimensional components (PC1, PC2, and PC3), which accounted for 98.6% of the variance. PC1 distinguished most of the evaluated variables and represented a greater proportion of the variation (95.1%), while PC2 (2.2%) and PC3 (1.3%) represented less of the percentage of the variance. The PCA revealed that the control, NK, and ML-NK10 groups were clustered on the left side and separated from the groups treated with ML (Figure 6B). Furthermore, the VIP revealed that MDA, AST, and ALP were the most relevant parameters in distinguishing ML-exposed animals from the other groups (Figure 6C).

### 3.6. Molecular Docking

**Figure 7:**
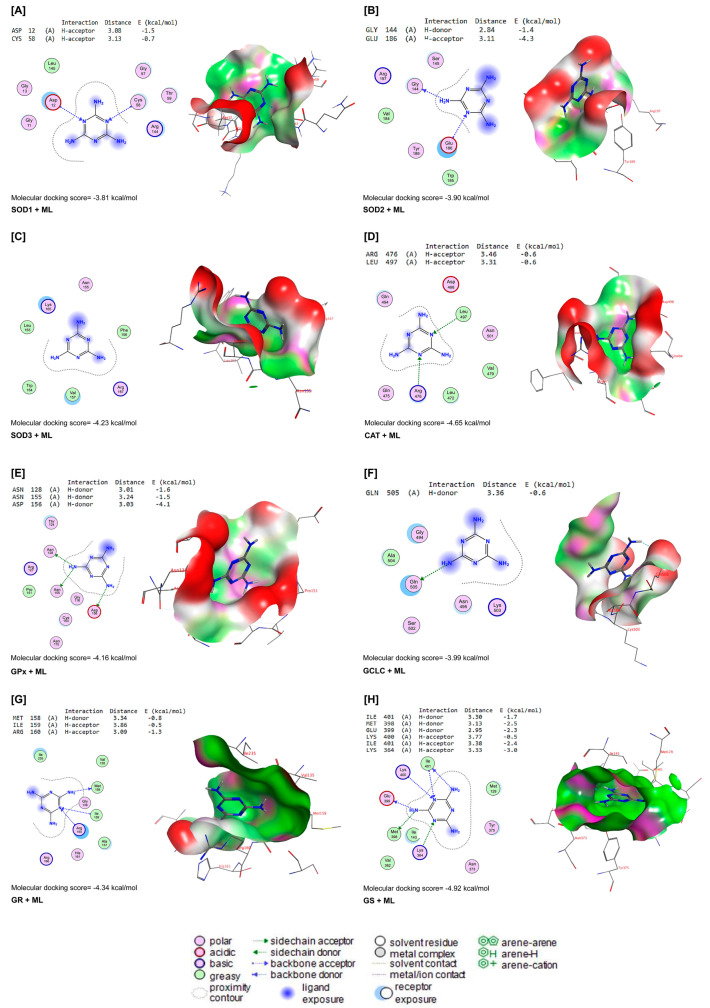
Molecular docking interactions of ML with rat (**A**) superoxide dismutase (SOD1), (**B**) SOD2, (**C**) SOD3, (**D**) catalase (CAT), (**E**) glutathione peroxidase-1 (GPx-1), (**F**) glutamate-cysteine ligase catalytic subunit (GCLC), (**G**) glutathione reductase (GR), and (**H**) glutathione synthetase (GS). The letter A in the parenthesis in each protein indicates the chain A of the target protein.

ML interacted with the binding sites of SOD1, SOD2, SOD3, CAT, GPx-1, GCLC, GR, and GS with binding energies of −3.81, −3.90, −4.23, −4.65, −4.16, −3.99, −4.34, and −4.92 kcal/mol, respectively (Figure 7A–H). ML bound with ASP12 and CYS58 in the binding site of SOD1 by H-acceptor bonds (Figure 7A). In the binding site of SOD2, ML was bound to the GLY144 with H-donors and GLU186 with H-acceptors (Figure 7B). Similarly, ML interacted with ARG467 and LEU497 residues in the binding site of CAT by H-acceptor bonds (Figure 7D). In the binding site of GPx-1, ML was bound by H-donors with ASN128, ASN155, and ASP156 residues (Figure 7E). Also, ML bound with GLM505 residues by H-donors in the binding site of GCLC (Figure 7F). In Figure 7G, ML interacted with MET158 by H-donors and with ILE159 and ARG160 by H-acceptor bonds in the binding site of GR. Furthermore, ML bound to the ILE401, MET398, and GLU399 residues by H-donors in the active site of GS, and interacted with LYS400, ILE401, and LYS364 residues by H-acceptor bonds (Figure 7H).

**Figure 8:**
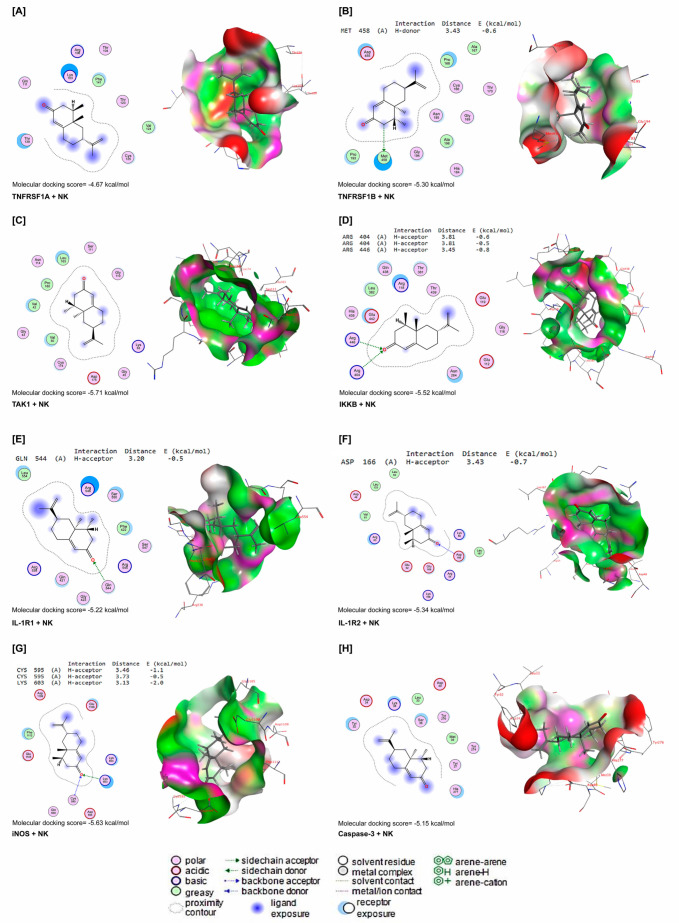
Molecular docking interactions of NK with rat (**A**) tumor necrosis factor receptor superfamily member 1A (TNFRSF1A), (**B**) tumor necrosis factor receptor-associated factor 1 B (TNFRSF1B), (**C**) transforming growth factor beta-activated kinase 1 (TAK1), (**D**) inhibitor of nuclear factor kappa-B kinase subunit beta (IKKB), (**E**) interleukin-1 receptor type 1 (IL-1R1), (**F**) interleukin-1 receptor type 1 (IL-1R2), (**G**) inducible nitric oxide synthase (iNOS), and (**H**) caspase-3. The letter A in the parenthesis in each protein indicates the chain A of the target protein.

NK interacted with energy values of −4.67, −5.30, −5.71, −5.52, −5.22, −5.34, −5.63, and −5.15 kcal/mol with TNFRSF1A, TNFRSF1B, TAK1, IKKB, IL-1R1, IL-1R2, iNOS, and caspase-3 binding sites (Figure 8A–H), respectively. NK interacted with the MET458 residue in the binding site of TNFRSF1B by H-donor bonds (Figure 8B). In Figure 2D, NK was bound with ARG404 and ARG446 residues in the binding site of IKKB by three H-acceptor bonds. In the binding site of IL-1R1, NK interacted with the GLN544 residue by H-acceptor bonds (Figure 8E). Furthermore, NK was bound with the ASP166 residue in the binding site of IL-1R2 by H-acceptors (Figure 8F). In addition, by H-acceptor bonds, NK interacted with CYC595 and LYS603 residues in the binding site of iNOS (Figure 8G).

## 4. Discussion

ML is a common pollutant and carcinogen that threatens human and animal health, and has sparked major global concerns [29]. It is widely dispersed in the environment including water and soil [30], and has been incriminated as a substance in food adulteration [31]. The liver is the primary organ for metabolism and detoxification and one of the organs most seriously affected by ML exposure [10,29]. 

Ample evidence strongly indicates that LPO, cellular antioxidant insufficiency, and disruption of mitochondria are key pathogenic pathways associated with ML exposure [32]. Accordingly, this study revealed increased LPO and oxidative stress following ML exposure, as indicated by increased MDA and NO levels accompanied by a considerable reduction in CAT and SOD activities and GSH levels. NO is a free radical; at higher levels, it perturbs the mitochondrial electron transport chain, which increases e^−^ loss and produces significant quantities of O_2_^−^ [33]. Furthermore, O_2_^−^ and NO radicals react together, resulting in the generation of nitrogen radical species that cause oxidative stress and have deleterious consequences on living cells. Interestingly, SOD, an endogenous antioxidant enzyme, functions as the first line of enzymatic antioxidant defense necessary for the dismutation of O_2_^−^ to O_2_ and H_2_O_2_ [32,34]. Notably, CAT is a crucial enzyme for the conversion of hydrogen peroxide into oxygen and water vapor [24,35]. Alternatively, Fenton’s reaction is encouraged when ML-generated ROS, which exhausts the antioxidant enzymes, leads to the formation of abundant quantities of OH^•^, the most detrimental radical, because it degrades membrane lipids resulting in increased LPO, as indicated by elevated MDA levels [24]. More importantly, MDA itself has the potential to damage cellular proteins, DNA, and mitochondrial lipid membranes. It also might cross cell membranes easily and interact with intracellular molecules, which results in the propagation of cellular injury [12]. 

According to our findings and those of others [11,13], hepatic oxidative damage following ML exposure is triggered by increased ROS generation and liver antioxidant enzyme exhaustion. These findings were further supported by immunohistochemistry results that revealed the downregulation of Nrf2 expression in hepatic tissues after ML exposure. In addition, this finding was in agreement with the former study conducted by Wu et al. [36], who reported that Nrf2, a key modulator of the detoxification pathway, was decreased after ML exposure. Additionally, the molecular docking analysis demonstrated the interaction of ML with antioxidant enzymes, including SOD1, SOD2, SOD3, CAT, GPx-1, GR, GCLC, and GS in the docking pathway. 

Expectedly, the enhanced LPO and disruption of hepatic cell membranes detected following ML exposure might have enhanced the release of liver enzymes (AST, ALT, and ALP) into the circulation, resulting in increased levels in the serum, as observed in the current study. These results corroborate our earlier reports that demonstrated a positive association between MDA levels and elevated levels of hepatic enzymes [12,14,15]. El Rabey et al. [37], Early et al. [38], and Ahmed et al. [14] also reported significant hepatocellular membrane degradation with enhanced hepatic enzyme activities that confirmed significant impairment of liver function after ML exposure. These findings are in agreement with our histoarchitectural results, as we observed substantial pathologic alterations in liver tissues, including severe damage to hepatocyte membranes. In addition, researchers [29] reported an accumulation of hepatic aggregates and disruption of hepatic structure following ML exposure. This observation was also reported in previous studies [2,10,11], demonstrating that ML clearly disrupts rat liver tissue. 

Besides LPO and oxidative damage, ML-induced hepatic injuries have been associated with increased production of pro-inflammatory cytokines and significant inflammatory cell mechanisms involved in ML hepatotoxicity [39]. This finding aligns with our histopathological results, which elucidate inflammatory cell infiltration, and with our immunohistochemistry findings that exhibited increased expression of NF-κB in liver tissue. Intriguingly, NF-κB, a marker of the inflammatory process, is a transcription factor concerned with proinflammatory mediator regulation, chemokine expression, and cytokine activation [40,41,42,43,44]. In addition, the knockdown of Nrf2 can enhance NF-κB expression and cytokine production [45]. In the same manner, Kuo et al. [39] have documented upregulation of NF-κB expression in ML-intoxicated rats.

NK is a naturally occurring sesquiterpenoid, found in grapefruit peels and can be synthetically produced through biotransformation of valencene [27]. NK has a range of biological activities and is frequently utilized in cosmetics, food additives, and pharmaceutics [35]. Intriguingly, in the present study, liver function in ML-exposed rats was dramatically improved by NK remediation with either a low or high dose. The improvements included restoration of the aberrant serum levels of ALT, AST, and ALP to near-normal levels. Our findings corroborate earlier research showing that NK abolished d-galactosamine-stimulated acute liver damage in mice in a dose-dependent manner [26]. 

Overwhelming evidence confirms the idea that NK supplementation boosts antioxidant enzyme activity by modulating the expression of oxidative stress biomarkers, such as Nrf2 [46], along with decreasing LPO [24]. In support, our data revealed that NK remedy conferred robust antioxidant activity against ML-stimulated oxidative damage, proven by recovering the normal MDA and GSH levels together with CAT and SOD activities in the ML-treated rats, in a dose-dependent manner. Mechanistically, NK has the pre-emptive potential to capture free radicals and prevent cellular damage such as LPO and protein oxidation ascribed to its bioactive antioxidant ingredient: sesquiterpene ketone [46]. Similarly, Majid [35] and Park et al. [27] demonstrated that NK supplementation could protect the cell membrane from LPO and oxidative stress via increasing the activities of antioxidant enzymes. 

Besides its antioxidant activity, NK has demonstrated robust anti-inflammatory activity, which might be partly ascribed to the suppression of the NF-κB pathway [47], and IL1-β, IL-6, and TNF-α production [24] as well as possible reduction in cyclooxygenase-2 [46] and iNOS activities [27]. The current investigation also proposed that NK had anti-inflammatory properties in ML-exposed animals, which was confirmed by a substantial reduction in the lymphocytic infiltration and NF-κB expression levels in liver cells in a dose-dependent manner. Our data are in agreement with prior findings showing that NK mitigated carbon tetrachloride-induced hepatotoxicity in mice in a dose-dependent manner [24]. An additional report demonstrated that mice supplemented with NK exhibited enhanced antioxidant enzyme activity in their kidneys [48]. Interestingly, the molecular docking analysis demonstrated that NK interacted strongly with inflammation and apoptosis regulatory proteins (TNFRSF1A, TNFRSF1B, TAK1, IKKB, IL-1R1, IL-1R2, caspase-3, and iNOS). Taken together, these data reveal that NK supplementation might have hepato-protective effects by reducing the inflammatory reactions and apoptosis in ML-injured livers.

**Figure 9:**
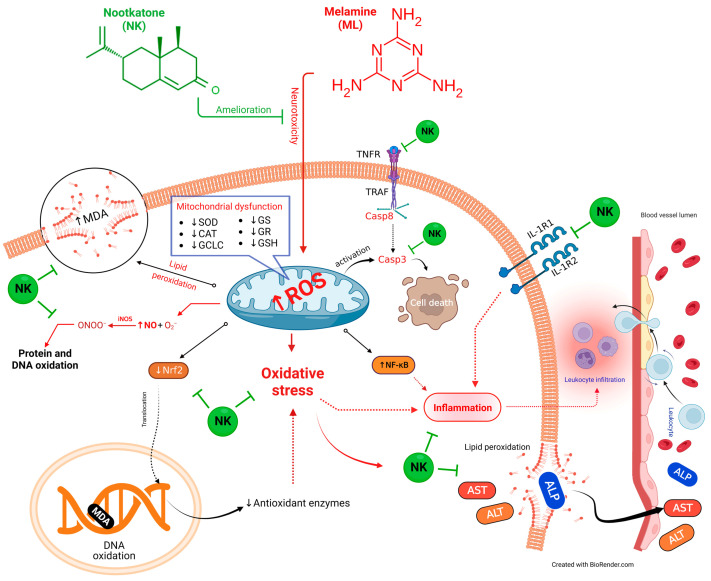
The protective impact of NK on ML-induced liver impairment is based on molecular mechanisms.

We performed multivariate statistical analysis, depicted by PCA, to assess the multiple contributions from different treatments on liver tissue. Each treatment was primarily identified on the PC1 axis (95.1%). ML-exposed animals were distinguished from the other treated groups because they clustered on the left side, apart from other treatment groups. The ML-NK10 co-treated group, on the other hand, was located close to the control and NK groups. In contrast to the other treatment groups, the clustering heatmap exhibited variations in all variable levels with respect to ML exposure. Thus, these findings robustly emphasize the potential protective effect of NK on ML toxicity. Figure 9 highlights the cellular mechanisms behind the protective impact of NK upon ML exposure. Based on these observations, we suggest that co-treatment with NK could mitigate ML-induced liver damage in a dose-dependent manner.

## 5. Conclusions

Taken together, our results revealed that the liver is one of the principal target organs for ML toxicity. ML exposure caused substantial hepatocellular injury which is attributed to increased oxidative stress, LPO, and inflammatory reactions. In a dose-dependent manner, NK supplementation ameliorated the ML-inflicted oxidative and inflammatory changes in the liver tissue. NK corrected biomarkers of hepatic toxicity and relieved oxidative stress and inflammation induced by ML. This hepatoprotective impact of NK is ascribed to its antioxidant and anti-inflammatory properties, along with its potential to restore the normal structure of the injured liver tissues. Molecular docking dynamics confirmed the anti-inflammatory and antioxidant proteins targeted by NK and ML. We thereby propose that NK supplementation could be an effective preventive approach for diseases or toxicants that trigger hepatic degeneration.

## Data Availability

The corresponding authors can provide the data used to verify the findings of this research upon request.
